# Early Decomposition of Retained Heavy Silicone Oil Droplets

**Published:** 2012-01

**Authors:** Touka Banaee

**Affiliations:** Eye Research Center, Khatam-al-Anbia Hospital, Faculty of Medicine, Mashhad University of Medical Sciences, Mashhad, Iran

**Keywords:** Heavy Silicone Oil, Vitrectomy, Tamponade, Chemical, Toxicity

## Abstract

**Purpose:**

To report a case of early decomposition of retained heavy silicone oil droplets.

**Case Report:**

The single highly myopic eye of a 16-year-old boy with history of scleral buckling and buckle revision developed redetachment due to inferior retinal dialysis. The patient underwent pars plana vitrectomy and injection of heavy silicone oil. Early emulsification of the silicone oil was observed following surgery, which was removed 4 weeks later in another operation. Retained heavy silicone droplets lost their heavier- than-water specific gravity within 2 months together with extensive iris depigmentation, and release of pigment granules into the anterior chamber and vitreous cavity.

**Conclusion:**

This case report demonstrates that heavy silicone oil droplets can undergo *in vivo* chemical decomposition with possible toxic effects on ocular tissues.

## INTRODUCTION

Heavier-than-water long acting internal tamponading agents have long been awaited by the vitreoretinal community for treatment of inferior retinal breaks. Densiron 68^1^, with specific gravity of 1.06 g/cm^3^ and viscosity of 1487mPa, is one of the latest generations of such agents licensed for use in Europe. Emulsification, cataract, glaucoma, excessive postoperative inflammation and retinal gliosis are among the complications described following the use of Densiron.^1^,^2^ Herein, we present a case in whom chemical decomposition of retained heavy silicone oil droplets within the eye caused extensive iris depigmentation.

## CASE REPORT

The single eye of a 16-year-old boy with history of scleral buckling and buckle revision developed inferior redetachment approaching the fovea. Pars plana deep vitrectomy with injection of heavy silicone oil (Densiron 68, Fluoron GmbH, Neu-Ulm, Germany) was scheduled for this large, myopic eye which also had a mild posterior subcapsular cataract.

The procedure included pars plana lensectomy, deep vitrectomy, injection of perfluorocarbon liquid (DK Line; Bausch and Lomb Inc., Waterford, Ireland), endolaser photocoagulation, superior peripheral iridotomy, air-fluid exchange and injection of 10 milliliters of Densiron 68 into the air filled vitreous cavity; all of which were accomplished without complications.

The retina was reattached after surgery but the postoperative course was complicated by an intense inflammatory response, early emulsification of silicone oil in the first week, and formation of macular pucker. Repeat pars plana vitrectomy along with epiretinal membrane removal and silicone oil injection was performed 4 weeks after the initial vitrectomy. Despite attempting meticulous removal of heavy silicone oil droplets, many remained in the eye and were obviously heavier than water in the early postoperative period, but became lighter with time.

**Figure 1.**
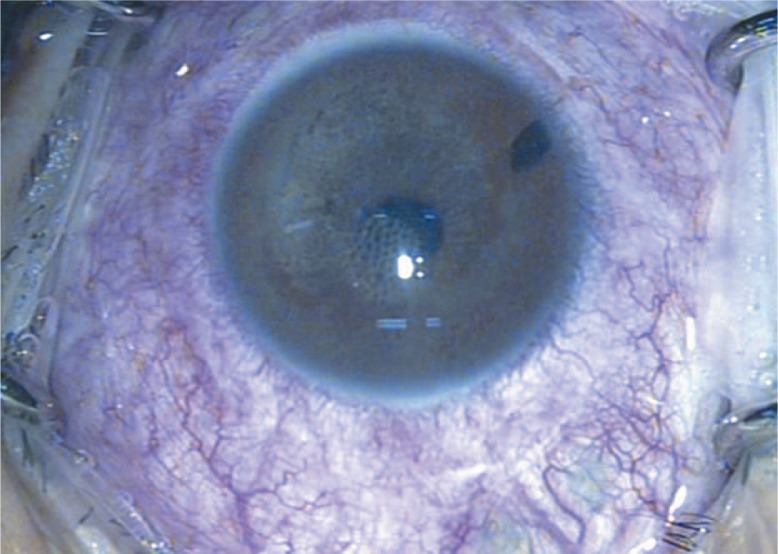
Diffuse iris depigmentation together with pigment globules on the iris and also floating in the anterior chamber, 3.5 months after injection and 2 months after removal of heavy silicone oil.

Two months after silicone oil removal, some droplets were visible in the mid-vitreous cavity, some adhered to the cornea in supine position while others still rested on the retinal surface. Along with these changes, the media became hazy and diffuse iris depigmentation developed together with pigment globules on the iris, and in the anterior chamber and vitreous cavity (Fig. 1).

**Figure 2.**
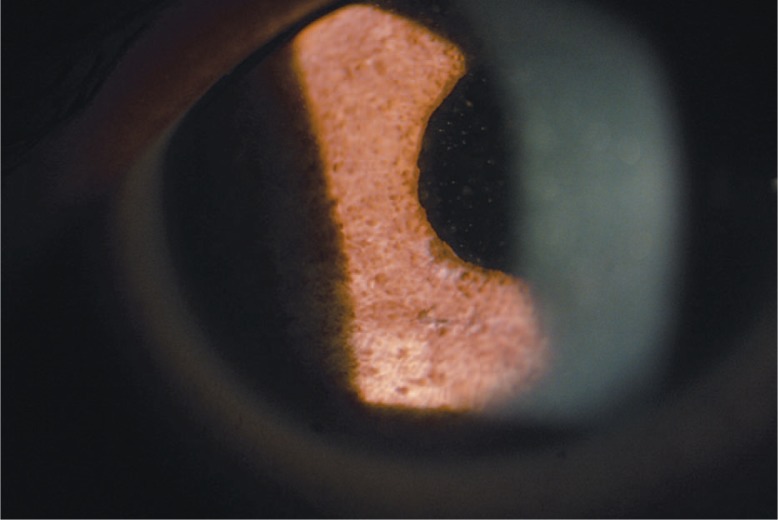
Intraoperative view of the eye during the last surgery demonstrates large, lighter-than-water silicone oil globules behind the cornea.

With time, increasing droplets were noted floating in the vitreous cavity, hindering fundus visibility and reducing corrected visual acuity from 20/100 to 20/200. Another washout surgery was performed 6 months later. Figure 2, taken at the time of this operation, demonstrates the large, lighter-than-water silicone oil droplets behind the cornea.

This surgery was successful in partially clearing the media and returning 20/100 vision to the patient.

## DISCUSSION

Excessive postoperative inflammation and epiretinal membrane formation are previously reported complications of Densiron^1^ which were observed in our patient. Early emulsification of Densiron has also been previously described.^1^ Large globe volume in this patient with some degree of under-filling and severe postoperative inflammation may have contributed to this complication.

Silicone removal was performed 4 weeks after initial surgery in this case. Although earlier removal could have been beneficial, considering the monocular status of this young patient and history of multiple procedures forced us to allow some time for resolution of postoperative inflammation in order to minimize the risk of proliferative vitreoretinopathy. Earlier removal may have resulted in fewer remaining silicone droplets but would not have prevented it altogether since emulsification had already begun and was severe one week after initial surgery.

The remarkable phenomenon observed in this patient was that the specific gravity of retained heavy silicone oil droplets in the eye may vary with time. Chemical decomposition of heavy silicone oil due to dissociation of perfluorohexyloctane from silicone oil appears a plausible explanation for this condition which occurred approximately 3.5 months after oil injection in our patient. Diffuse iris depigmentation in this eye, may be a toxic side effect of such chemical decomposition.

Pigment dispersion and phagocytosis of iris pigments along with pigmented keratic precipitates have previously been reported with regular silicone oil^3^,^4^, but diffuse iris depigmentation is an exaggerated response probably unique to the situation described in this patient. It seems that heavy silicone droplets may adsorb iris pigment granules due to the chemical characteristics they acquire after decomposition, transforming into pigmented globules in the eye. Chronic inflammation may be another explanation for these findings but no signs of chronic inflammation, including keratic precipitates or anterior chamber reaction out of proportion to that expected were seen.

This case re-emphasizes the possibility of early emulsification with the use of low viscosity heavy silicone oil in large eyes, and also provides clues to chemical decomposition of heavy oil droplets within the eye. Diffuse iris depigmentation in this case may be considered a previously unreported complication of heavy silicone oil.
